# Evaluation of nursing snippet-based intervention on parents’ burden and self-efficacy towards caring for their children undergoing limb lengthening surgery

**DOI:** 10.1186/s12912-026-04931-2

**Published:** 2026-07-04

**Authors:** Rawia Abd El-Ghany Mohamed, Nora Abd El-Alim Ebrahim, Hanan Elsayed Metwally

**Affiliations:** Pediatric Nursing, Faculty of Nursing, Benha University, Benha, Egypt

**Keywords:** Children, Limb lengthening surgery, Nursing snippet-based intervention, Parents’ burden, Self-efficacy

## Abstract

**Background:**

The intricacy of pin-site care and fixator modification makes caring for children having limb lengthening surgery extremely taxing on parents. Their self-efficacy, which is essential for positive postoperative results, is frequently lowered by this stress. Traditional teaching approaches might not be able to offer the ongoing assistance required to preserve caregiver confidence and lower stress levels.

**Aim:**

To examine the nursing microlearning (snippet-based) intervention on parents’ burden and self-efficacy in caring for their children undergoing limb lengthening surgery.

**Methods:**

The present study was carried out utilizing a one-group pretest–posttest research design. Setting: The present study was conducted at the inpatient pediatric surgical department, at Benha Specialized Pediatric Hospital in Benha city. Study sample: A purposive sample of 55 parents accompanying their children undergoing limb lengthening surgery. Study tools: Five tools were used for data collection, Tool I: A structured interviewing questionnaire. Tool II: Child medical data. Tool III: Parents’ reported practices checklist. Tool IV: Caregiver Self-Efficacy Scale. Tool V: Burden interviewing questionnaire.

**Results:**

Satisfactory knowledge increased from 16.4% pre-intervention to 87.3% post-intervention (*p* < 0.001), and competent practice increased from 21.8% to 89.1%. In addition, the proportion of parents with high self-efficacy increased from 10.4% to 41.7% (*p* < 0.001), while the proportion experiencing low parental burden decreased from 16.4% to 5.5%.

**Conclusion:**

The nursing snippet-based intervention may be associated with improvements in parents’ knowledge, practice, and self-efficacy and reducing parental burden. Integrating microlearning into routine pediatric orthopedic discharge education may support continuity of care beyond the hospital setting.

**Clinical trial number:**

Not applicable.

## Introduction

Limb lengthening surgery is one of the possible elongation procedures and represents a challenging pathway burdened by a high rate of complications. Additionally, this operation is still the only viable way for children with achondroplasia to restore body proportions more in line with the regular population and reach a stature comparable to the normal population range at maturity [1].

The functional and cosmetic consequences of limb deformity and shortening necessitate surgery. While more English-language publications support the practice of treating lower limb deformities and extending bones, fewer articles discuss upper extremity lengthening. Dick and Tietjen reported the first instance of humeral lengthening in 1978 [2].

Limb length disparity, a condition where one limb is shorter than the other owing to congenital, developmental, or post-traumatic factors, can be corrected with limb lengthening, a crucial orthopedic operation. Children who have limb lengthening surgery might improve their psychological and emotional well-being while gaining more independence in everyday activities like sports, public transportation, and personal cleanliness [3].

Limb lengthening with external fixation can correct the lower extremity deformity [4]. While, internal lengthening devices became more popular than external fixators. This was attributed to their improvement and lower complication rates [5].

The care of children undergoing surgery places a substantial burden on parents, both emotionally and in terms of practical caregiving responsibilities. The level of burden of care varies depending on the type, intensity and duration of the disease of the person receiving the care. Furthermore, giving care to a sick child, monitoring the treatment and medications used, meeting the child’s nutrition and personal care needs, and hospitalizations affecting family integrity adversely affect parents’ lifestyle and further deepen the difficulties they experience [6].

The suffering that a family member’s primary caregiver experiences, including consequences for their health, psychological well-being, finances, and social life, is referred to as caregiver’s load. In this context, a caregiver is someone who gives unpaid care to a sick child and who has a relationship with the child, either biologically or by friendship. Mothers, fathers, grandmothers, and aunts are typically considered caregivers [7].

Family caregiver burden is a complicated reaction to the financial, social, emotional, psychological, and physical strains of providing care. The biological, psychological, sociological, ethnic, cultural, and religious facets of the life of formal caregivers are all changeed by this complex process [8]. Understanding the caring burden, stress, and personal resources of parents is crucial. The child’s chronic illness affects both parents. In terms of personal requirements, mothers and fathers have disadvantages as compared to parents of healthy children [9, 10].

Self-efficacy describes how respond to stressful events in life and is only used in the care sector to explain how caregivers deal with persistent difficulties [11].

In the context of pediatric surgery and long-term care, parents frequently take on the role of primary caregivers and bear heavy responsibilities in managing their child’s recuperation and daily requirements. Additionally, improving parental understanding may boost parental efficacy and confidence. Therefore, nurses should be able to comprehend the emotional and psychological responses of the parents, as well as establish a great learning atmosphere and show crucial interest and support [12].

Educating families of children having surgery has been the subject of numerous research. Numerous techniques have been linked to lowering anxiety in children and their relatives. Strong interpersonal interactions between hospital staff and parents/children, emphasizing rapport, communication, and honesty, as well as education for parents/children. One method of preparing parents for the stress levels encountered during a preoperative period is through interventions [13].

The use of microlearning in education has been shown to improve learners’ knowledge and self-assurance. Health professions educators support microlearning as a way to support parent education, training, and continuing education. Microlearning is the acquisition of knowledge or skills in the form of small units [14].

A nursing snippet-based intervention is a cutting-edge method of instruction that presents material in brief, targeted chunks, or “snippets.” When used in parent education, this approach may improve and engagement by giving parents short, planned learning units that fit into their everyday schedules. The idea is based on micro-learning theory, which prioritizes brief, focused learning opportunities over lengthy class periods [15].

Additionally, short modules and instructional films have been shown to be successful in enhancing parental scaffolding abilities and self-efficacy, allowing parents to better assist their children’s learning and healing processes [16]. Thus, snippet-based instruction offers a productive, parent-centered framework that may support the parent’s role as an improve caregiver and educator, encourages active learning, and lessens cognitive overload [17].

Parents of children undergoing limb lengthening surgery are exposed to complex and stressful care demands, which may increase their cognitive load and learning burden. Traditional educational approaches may be insufficient in addressing these challenges effectively. Therefore, there is a growing need for concise, accessible, and easily digestible educational strategies such as microlearning and snippet-based interventions to enhance parental understanding and support association caregiving [18].

### Significance of the study

Surgery has been linked to anxiety and severe psychological reactions in children and parents due to concerns about anesthesia, fears of cancer or death, and anticipated pain and physical discomfort. Even in surgeries that doctors deem “minor,” these have been shown to negatively change surgical procedures and the patient’s recuperation. Given the evident yet crucial role parents play in managing children with surgical conditions, it would be pertinent to understand the caregiver burden [7].

Children’s surgeries are known to be stressful experiences for both the child and the family. Expectations, views of the sickness, and the healing process all contribute to the high level of parental stress that persists during a child’s hospital stay. Additionally, both children and their parents may have unpleasant and challenging experiences as a result of pediatric surgery. “Burden of care” refers to the negative effects of a caregiver’s caregiving, including physiological, psychological, economic, social, and familial issues [6].

For those with achondroplasia, the most prevalent skeletal dysplasia with an estimated prevalence of 1:22,000 live births and over 250,000 affected individuals globally, limb lengthening surgery is a controversial option. The combined prevalence of achondroplasia in Europe is 3.5 per 100,000 live births. Impaired endochondral bone formation is the outcome of a heterozygous pathogenic mutation in the Fibroblast Growth Factor Receptor 3 gene (FGFR3) [19, 20].

Rehabilitation and postoperative care are mostly the responsibility of the parents of children undergoing surgery. Research indicates that a high caregiver burden might have a detrimental effect on child outcomes by increasing parental stress and decreasing adherence to care routines. On the other hand, parental self-efficacy-boosting therapies greatly lessen load and promote the wellbeing of both parents and children [7].

### Theoretical framework

The theoretical framework of this study was developed to explain how a snippet-based teaching strategy can influence parents’ burden and self-efficacy while caring for their children undergoing limb lengthening surgery. Parents of children who undergo this complex surgical procedure often experience high levels of physical, emotional, and psychological burden due to the long treatment period, intensive care requirements, and fear of complications. At the same time, their ability to cope and provide effective care depends largely on their level of knowledge, skills, and confidence.

Current study is guided by Bandura’s Self-Efficacy Theory [21–23] supported by Lazarus and Folkman’s Stress and Coping Theory [24, 25]. According to the concept, parents perceived self-efficacy is a key factor in evaluating their capacity to manage the caring responsibilities related to limb lengthening surgery. Increased parental self-efficacy improves coping mechanisms and caregiving techniques, which lessens the perceived burden of parenting. On the other hand, parents’ self-efficacy may be severely changed and their burden may grow due to increased caregiving obligations and child-related clinical variables.

The high technical complexity of care, which includes frame modifications and careful pin-site care, frequently overwhelms parents of children following limb-lengthening surgery. Due to this intricacy, parents may experience “cognitive overload” and elevated anxiety as a result of the amount of medical knowledge exceeding their mental processing capabilities [26–28]. By giving too much material at once, traditional long-form teaching approaches may unintentionally make this problem worse. As a manageable digital lifeline for parents in high-stress surgical environments, microlearning through snippet-based interventions, on the other hand, provides a strategically superior solution. By delivering information in small, digestible, and focused units, it reduces unnecessary cognitive load and improves the retention of critical caregiving skills.

### Methodology

#### Research design

The present study utilized a one-group pretest–posttest design to evaluate immediate pre–post differences in parents’ burden and self-efficacy among parents of children undergoing limb lengthening surgery who were exposed to a snippet-based educational approach.

This study aimed to evaluate the following three study propositions:

1. Parents who are exposed to the snippet-based intervention show differences in knowledge and practice scores between post-intervention and pre-intervention.
2. Parents’ burden scores show differences between post-intervention and pre-intervention measurements.
3. Parents’ self-efficacy scores may differ between post-intervention and pre-intervention measurements.

### Sample

A purposive sample of 55 parents accompanying their children undergoing limb lengthening surgery was recruited from the pediatric orthopedic units at the previously mentioned setting. Data were collected over a six-month period across morning and afternoon shifts. The one-group pretest–posttest design was selected due to ethical considerations related to providing the educational intervention to all participating parents.

- The sample size was calculated by the following formula: *n* = N/1+N(e)^2^ [29] Where;

- **n = sample size=** 55
- **N = Total population size=** 64

- parents

- **e = Margin of error=** 0.05
- Therefore, **sample size (n) =**

**64/ (1+64 × 0.0025) = 55.1**.

Despite the sample size being estimated using Slovin’s technique, a post-hoc power analysis showed that the sample (*n* = 55) was sufficient to detect high effect sizes. They were taken according to the following:

**Inclusion criteria for children:**

- From both genders.
- Children who have already undergone limb lengthening surgery.
- Ages ranged from 5-12 years.
- Free from any other previous leg surgeries.

**Exclusion criteria for children:**

- Children with cognitive or developmental delays that prevent understanding of instructions

**Inclusion criteria for parents**

- Willing to participate in the snippet-based teaching strategy sessions.
- Parents who own a smartphone and are able to access digital teaching materials.

**Exclusion criteria for parents**

- Parents with psychiatric or cognitive conditions that may interfere with participation

### Setting

The current study was conducted at the inpatient pediatric surgical unit at Benha Specialized Pediatric Hospital affiliated to Ministry of Health in Benha City. It included two pediatric surgical departments that were located at Building B of the hospital on the second and third floors, each department comprises five rooms, each room contains four beds, and the recovery room contains two beds.

### Tools of data collection

Five tools were used to obtain data relevant to the current study. It was prepared in simple Arabic language. Each parent was interviewed exclusively for filling the knowledge questionnaire sheet. These included the following tools:

#### Tool (I): a structured interview questionnaire

This tool was developed by the researchers after reviewing the scientific and relevant literature. That was written in an Arabic and consisted of two parts:

*Part (1): Parents’ characteristics such as;* age, level of education, occupation, residence, consanguinity relation between parents and attendance of training courses regarding the care of their children undergoing limb lengthening surgery.

*Part (2): Parents’ knowledge* regarding limb lengthening surgery. It was designed by the researchers in the light of related studies and researches [30, 31]. It contained (31 questions) in the form of multiple choice and true/false, which included definition and purpose of limb lengthening (2 questions), risks/benefits of surgery(2 questions), types of devices used (1 question), importance of preoperative medical evaluation and investigations(3 questions), role of the parent in preparing the child physically and psychologically(4 questions), expected duration of the lengthening and consolidation phases (2 questions), postoperative Care (7 questions), signs of complications (1 questions), home care and follow-up (5 questions), and expected outcomes (4 questions).

##### Scoring system

The parents’ knowledge was checked with the model answer after completing the interview questionnaire. The correct answers scored (1), and the incorrect or do not know answers scored (0). The total degree ranges from 0 to 31 (31 questions). The parents’ total knowledge was classified as the following:

- ≥60% was considered a satisfactory level of knowledge
- <60% was considered an unsatisfactory level of knowledge.

#### Tool (II): child medical data

It was developed by the researchers and consisted of two parts as the follow:

*Part 1:*

- Personal characteristics of the studied children, it included age, gender, and child rank.

*Part 2:*

- Medical history of children, it included medical diagnosis, complications that occurred as a result of limb lengthening surgery.

#### Tool (III): parents’ reported practices checklist

It was adapted from [32–35]; to assess parents’ reported practice towards care of their children after limb lengthening surgery. It includes 11 main items and the total steps were 65 steps including; **immediate post operative care** (6 steps), **pain and analgesia management** (5 steps), **essential wound care/ daily cleaning of pin sites** (8 steps), **skin and personal hygiene management** (6 steps), **emotional/psychosocial support** (4 steps), **assist with mobility and safe transfers** (6 steps), **home environment & equipment management** (7 steps), **device/frame care & adjustments** (7 steps), **physiotherapy / range-of-motion exercises** (5 steps), **monitoring for complications & early reporting** (7 steps), **adherence to follow-up schedule after surgery** (4 steps). Score [1] was given to a correctly done step. Score (0) was given to incorrectly done or not done step. The total steps included 65 steps.

##### Scoring system for parents’ reported practice

Total scores were ranged from (0–65). Accordingly, parents’ reported practices were classified as the following:

- Less than 85% was considered incompetent practice level
- Equal to or more than 85% was considered competent practice level

The cutoff points for knowledge and practice were determined based on a percentage scoring system commonly used in nursing and health research [36]. This approach has been widely adopted in previous studies to categorize levels of competency and facilitate interpretation and comparison of findings.

#### Tool IV: caregiver self-efficacy scale (CSES) adopted from [37]

It was written in an Arabic language and consisted of 15 items measuring confidence in managing caregiving tasks and obtaining respite, within 3 subscales (self-efficacy for obtaining respite /support, self-efficacy for responding to child’s distress/disruptive behaviors, and self-efficacy for controlling upsetting caregiving-related thoughts).

##### Scoring system for caregiver self-efficacy scale

0–100 confidence rating scale, classified as 0–33.3% considered Low self-efficacy, 33.4%-66.6% considered Moderate self-efficacy, and 66.7%-100% considered High self-efficacy.

#### Tool V: burden interviewing questionnaire

Modified version of Zarit Burden Interviewing questionnaire (ZBI) developed by [38]. This tool was used to measure the extent of family caregiver have physical, social and psychological burden. The ZBI questionnaire consisted of 22 items Burden in the relationship 6 items, Emotional wellbeing 7 items, Social and family life 4 items, Finances 1 items, and Loss of control over one’s life 4 items.

Categorized in five main sections: 5-point Likert scale of response for each statement indicates how often the caregiver feels that way: (0) never, (1) rarely, (2) sometimes, (3) quiet frequently, nearly always (4). The scale scoring system: The burden level was rated from (0–20) little burden, (21–40) mild burden, from (41–60) moderate burden, from (61–88) high burden.

It aims to assess the level of burden experienced by the principal caregivers of older persons with dementia and disabled persons. The ZBI provides an assessment of subjective burden, that is, the appraisals caregivers make of the change that providing care has on their lives. Subjective appraisals are a fundamental dimension in understanding the stress process. Measures of the patients’ symptoms or problems in functioning and behavior provide a context in which burden is experienced, but caregivers’ subjective experiences of the change of problems on their lives can vary considerably. Subjective burden as measured by the ZBI is a critical dimension, because caregivers’ perceptions of the change of providing care on their lives will affect their actions and the decisions they make, such as about seeking help or continuing in the care role.

### Preparatory phase

In order to become more familiar with the various parts of the study, this phase involved reviewing pertinent local and worldwide literature as well as recent studies. It also involved constructing the study’s tools using journals, scientific books, magazines, and evidence-based articles.

### Tools validity and reliability

A panel of three independent experts of pediatric nursing specialists from Benha University’s Faculty of Nursing evaluated the study tools’ validity in order to assess the items’ clarity, relevance, and applicability. The changes were made to guarantee their accuracy and applicability. The Cronbach’s alpha test was used to determine the measures’ internal consistency. It was 0.85 for practice and 0.78 for knowledge.

### Pilot study

It was carried out on six parents, or 10% of the study participants, in order to assess the study tools’ applicability and calculate the time needed to finish them. No significant changes were made to the study instruments in light of the pilot study’s findings. As a result, the study sample was expanded to include the parents from the pilot study.

### Procedure for data collection

The researchers were found in the study setting by using rotation two days per week (Saturday and Wednesday) during the morning shift to gather data using the previously mentioned tools. The fieldwork was completed over a six-months period.

The following phases were carried out to achieve the aim of the current study; assessment, planning, implementation and evaluation phases.

### Assessment phase

The researchers interviewed each parent individually; the aim and duration of the study were explained. Oral and written consents to participates in the study before data collection obtained.

### Planning phase

The researchers developed the nursing snippet-based intervention after reviewing the related literature and determined the needs in the assessment phase. The nursing snippet-based intervention utilized in the current study included three methods as following:

1. **Snippet videos:** Snippet videos are short, focused, and easily digestible video segments that deliver essential information in a concise and engaging way. They are particularly useful for educating parents about child care, medical procedures, rehabilitation, and health promotion.

- The duration of video Typically 5–7 minutes per video, focused on one topic or skill at a time, visual demonstrations (e.g., physiotherapy exercises, wound care). Also, videos can be accessed anytime, often via mobile device.
- Snippet had a great importance for parents through enhancing understanding because parents can focus on one concept at a time. Moreover, short videos are easier to remember than long lectures. Also, snippet videos increase engagement because videos with visuals and animations grab attention better than text.
- Parents can pause, replay, or share videos, allowing flexible learning. Furthermore, it supports practical skills and demonstrates procedures like bandage changing, physiotherapy exercises, or limb-lengthening care. Helps parents gain confidence in applying care techniques at home and feel less anxious when guided visually

2- **Snippet Infographics** are visual representations of information, or instructions that combine graphics, icons, charts, and brief text to make complex content easy to understand. Focus on a single concept or procedure per infographic, can be printed or shared digitally

#### Importance of infographics for parents

- It simplifies complex information by showing step-by-step instructions visually (e.g., limb care after surgery). Also, parents can keep a printed or digital copy for fast guidance at home.

3- **Snippet Single-Focus Modules** are short, focused teaching units that cover one specific skill or concept at a time using brief text explanations. It covers one topic per module, easy to share digitally or use in print, and supports better adherence to care instructions it was applied by using the following steps:

1. **One Skill per Snippet** – Focus on a single task.
2. **Show Then Explain** – Demonstrate first, then give 1–3 sentences summarizing steps.
3. **Use Simple Language** – Parent-friendly, avoid jargon.
4. **Use printed colored cards** summarizing critical steps (such as pain management, pin-site care, and danger signs) were also provided during hospital discharge
5. **Highlight Key Points** – Emphasize warnings or critical steps.
6. **Share Accessibly** –WhatsApp, or printed handouts.

### Implementation phase

The nursing snippet-based intervention was applied through short, focused instructional segments that covered essential aspects of their child’s limb lengthening care. These snippets were presented sequentially and reinforced through direct guidance. The researchers ensured consistent delivery, clarified questions, and supported parents’ skill development throughout the snippet teaching strategy implementation considering the appropriate level of Arabic language for the parents’ education.

- Session’s implementation schedule was established to suit parents including date, time, place, topics, and duration of all snippet sessions.
- There were eight meetings in all, lasting 40 to 50 minutes over the course of four months. Moreover, eight sessions containing the study objectives and carried out through (3 session for the theoretical as showed in Table A and 5 sessions for the practical part as showed in Table B) using different snippet teaching strategies. Ten groups of parents were created, with five to six parents in each group.

**Table A:** A nursing snippet-based intervention for theoretical part

Theoretical part	Snippet Video	Infographic / Visual	Snippet Single-Focus Modules (Brief Text Explanation)
Definition and Purpose of Limb Lengthening	Short animation explaining the concept of bone lengthening and how new bone forms.	Diagram showing bone segments, external fixator/device, and distraction process.	Clear text module defining limb lengthening, goals, and indications in simple language.
Risks and Benefits of Surgery	Video summarizing common risks (infection, pain, nerve irritation) and expected benefits (corrected deformity, improved function).	Risk–benefit comparison chart with icons.	Brief text listing major risks and benefits, with emphasis on parental understanding and early reporting.
Types of Devices Used	Demonstration video showing external fixators and internal lengthening nails.	Device comparison chart with labelled diagrams.	Step-by-step explanation of each device, its purpose, and how it works.
Importance of Preoperative Evaluation & Investigations	Video explaining required investigations (X-ray, blood work, anesthesia assessment).	Flowchart of preoperative assessment steps.	Module explaining why each investigation is needed and what parents should expect.
Role of the Parent in Preparing the Child Physically & Psychologically	Video showing parent–child interaction, preparation routines, and communication tips.	Checklist visual for psychological and physical preparation.	Short text guiding parents on how to reduce anxiety, encourage cooperation, and prepare physically (nutrition, hygiene, rest).
Expected Duration of Lengthening & Consolidation Phases	Visual timeline video showing each phase week by week.	Timeline graphic from surgery → distraction → consolidation → full healing.	Explanation of typical timeframes and what happens during each phase.
Postoperative Care (General)	Video on positioning, monitoring, and early home care.	Postoperative care flow diagram.	Simple instructions describing daily tasks parents must perform.
Signs of Complications	Scenario-based video showing early signs of infection or device issues.	Red-flag warning poster with visual icons.	Focused text describing when to seek emergency help.
Home Care & Follow-Up	Home-setting demonstration of daily routines, mobility support, and device care.	Home care checklist.	Clear explanation of home care responsibilities and follow-up visit schedule.
Expected Outcomes	Motivational video showing recovery progress and typical long-term outcomes.	Before/after visual comparison of expected limb improvement.	Text module outlining realistic outcomes and factors influencing success.

**Table B:** A nursing snippet-based intervention for practical part

Practical Task / Skill	Snippet Video	Infographic / Visual	Snippet Single-Focus Modules (Brief Text Explanation)
Immediate Postoperative Care	Demonstration video showing positioning, monitoring vital signs, limb elevation, and maintaining a safe environment.	Step-by-step flowchart for immediate postoperative care.	Brief text describing the essential steps of immediate postoperative care clearly and sequentially.
Pain and Analgesia Management	Video showing clear explanation of pain recognition, medication timing, monitoring improvement, and comfort measures. how to assess pain in children, administer prescribed analgesics, and document response.	Infographic showing step by step analgesia management.	“Give pain medications as prescribed. Keep child comfortable and in recommended position.”
Essential Wound Care	Video of step-by-step dressing removal & application	Labeled diagram of wound care steps & warnings	“Wear gloves, remove old dressing carefully, clean area, apply new dressing gently. Watch for redness or discharge.”
Daily Pin Site Cleaning	Video demonstrating aseptic pin-site cleaning using real equipment.	Visual guide with steps for pin-site care.	“Check screws and pins gently; do not force. Call doctor if pins feel loose or cause pain.”
Hand Hygiene	1–2 min video showing proper handwashing	Stepwise diagram of handwashing steps	“Wash hands with soap for 20 sec before care. Dry with a clean towel.”
Skin and Personal Hygiene Management	Video of safe bathing, skin inspection, and maintaining hygiene around device.	Hygiene checklist with essential tasks.	“Keep the skin clean and dry, bathe carefully around the device, and check for irritation or injury.”
Emotional / Psychosocial Support	Video demonstrating comforting techniques, parent–child communication, reassurance, and coping strategies after surgery.	Infographic showing emotional support tips parents should perform.	“Offer praise, calm voice, gentle touch. Encourage child to express feelings.”
Assist with Mobility & Safe Transfers	Video demonstration of safe bed-to-chair transfers, walking with aids, and protecting the operated limb.	Mobility infographic with safety rules. -Diagram of safe walking techniques	“Use assistive devices as instructed. Ensure clear floor and maintain balance.”.
Home Environment & Equipment Management	Short video illustrating safe space, removing hazards, and equipment placement.	Diagram of safe home layout	“Remove obstacles, keep floor dry, provide stable furniture to prevent falls.”.
Device / Frame Care & Adjustments	Step-by-step video demonstrating daily inspection and safe frame adjustments.	Diagram of device care with labeled parts.	“Clean and inspect device daily”
Physiotherapy / Range-of-Motion Exercises	Physiotherapist-led demonstration of Range-of-Motion exercises, stretching, and strengthening.	Exercise infographic with Range-of-Motion steps.	“Lift child’s leg slowly, bend knee gently, repeat 5 times per session, twice daily. Stop if painful.”.
Monitoring for Complications & Early Reporting	Scenario-based video showing early signs of infection, hardware problems, or neurovascular issues.	Red-flag infographic with warning signs requiring immediate reporting.	“Check for redness, swelling, pus, or fever. Contact doctor immediately if any appear.”
Adherence to Follow-Up Schedule After Surgery	Video explaining importance of regular clinic visits, X-rays, and frame adjustments.	Calendar-style visual showing follow-up timeline.	“Keep calendar of appointments. Attend all follow-ups to check progress and adjust care.”

### Evaluation phase (post nursing snippet-based intervention)

The parents’ burden, self-efficacy, knowledge and practice were immediately evaluated after a nursing snippet-based intervention and after 3 months of nursing snippet-based intervention. All questionnaires were administered to the same participants in a single session using the same data collection method. This approach may introduce common method bias, shared rater associations, social desirability bias, and potential respondent fatigue or order effects, which may influence the consistency and direction of responses.

#### Data analysis

Using (SPSS version 22), the data was coded and converted into a specifically created format for computer entry. Frequency, percentages, mean, and standard deviation were used as descriptive statistics. Additionally, chi-square was used to assess the study’s premise. At *p*-value ≤ 0.001, a highly significant difference was found; at *p*-value *p* ≤ 0.05, a statistical difference was found; and at *p*-value *p* > 0.05, no statistical difference was found.

## Results

Table 1 reveals the parents’ characteristics; it was found that, the mean age of the studied parents was 35.94 ± 3.52 years and the majority (87.3%) of them were mothers. According to parents’ educational level more than two thirds (69.1%) of them had university education. Additionally, it was noticed that, slightly less than two thirds (61.8%) of parents were unemployed and more than two thirds (67.3%) of them from rural area. Moreover, less than three quarters (70.9%) of the studied parent had no consanguinity relation and all (100%) of them not attended any previous training courses regarding limb lengthening surgery.

**Table 1:** Percentage distribution of the studied parents according to their personal characteristics (*n* = 55)

Parents’ characteristics	No.	%	
**Age in years**			
20->30	11	20.0	
30->40	28	50.9	
≥40	16	29.1	
Mean ±SD	35.94 ± 3.52		
**Gender**			
Fathers	7		12.7
Mothers	48		87.3
**Educational level**			
Intermediate education	11	20.0	
University education	38	69.1	
Post graduate education	6	10.9	
**Occupation**			
Yes	21	38.2	
No	34	61.8	
**Residence**			
Rural	37	67.3	
Urban	18	32.7	
**Consanguinity relation between parents**			
Yes	16	29.1	
No	39	70.9	
**Attendance of training courses regarding limb lengthening surgery**			
Yes	0	0.0	
No	55	100	

Table 2 shows children’s personal characteristics; it was observed that, the mean age of the studied children was 8.32 ± 2.53. As regards gender it was found that slightly less than two thirds (65.5%) of children were females. Additionally, it was noticed that, two fifth (40.0%) of the studied children was the first child in order.

**Table 2:** Percentage distribution of the studied children according to their personal characteristics (*n* = 55)

Children’s personal characteristics	(n = 55)	
	No.	%
**Age in years**		
5->9	31	56.4
9-≥12	24	43.6
x±SD	8.32 ± 2.53	
**Gender**		
Males	19	34.5
Females	36	65.5
**Ranking of the child**		
First	22	40.0
Second	14	25.5
Third	12	21.8
Fourth	7	12.7

Table 3 explains children’s medical history; it was found that less than half (43.6%) of them diagnosed with congenital short femur. Additionally, two quarters (40%) of children had complications as a result of limb lengthening surgery and less than half (45.5%) of children who had complications were complicated with joint stiffness.

**Table 3:** Percentage distribution of the studied children according to their medical history (*n* = 55)

Children’s medical history	(n = 55)	
	No.	%
**Medical diagnosis of children under limb lengthening surgery**		
Congenital short femur	24	**43.6**
Achondroplasia	15	27.4
Rotational deformity	11	20
Post traumatic damage	5	9
**Complications that occurred as a result of limb lengthening surgery**		
Yes	22	**40**
No	33	60
**If yes, the complications are (n = 22)**		
Pin- site infection	2	9.1
Muscle contractures	7	31.8
Joint stiffness	10	**45.5**
Deep venous thrombosis	3	13.6

Table 4 the majority (83.6%) of the studied parents had unsatisfactory knowledge at pre-test. This proportion changed to 87.3% and 78.2% of parents demonstrating satisfactory knowledge at immediate post-test and at 3-month follow-up, respectively.

**Table 4:** Percentage distribution of the studied parents’ total knowledge regarding limb lengthening surgery pre, immediately post and after 3 months of nursing snippet-based intervention (*n* = 55)

Items	**Pre a snippet-based teaching strategy implementation** (**n = 55)**		Immediately post a snippet-based teaching strategy implementation (n = 55)		After 3 months of a snippet-based teaching strategy implementation (n = 55)		X2(1)	p-value	X2(2)	p-value
	No.	%	No.	%	No.	%				
**Total knowledge level**							28.16	*p* < 0.001**	2.42	*p* > 0.05 ns
Satisfactory (≥60%)	9	16.4	48	**87.3**	43	**78.2**				
Unsatisfactory (<60%)	46	**83.6**	7	12.7	12	21.8				

** Highly statistical significant difference at *p* value ≤ 0.001

^ns^ No statistical significant difference at *p* value *p* > 0.05

X2(1) Difference between pre-test and post-test

X2(2) Difference between post-test and follow-up test

**Fig. 1:**
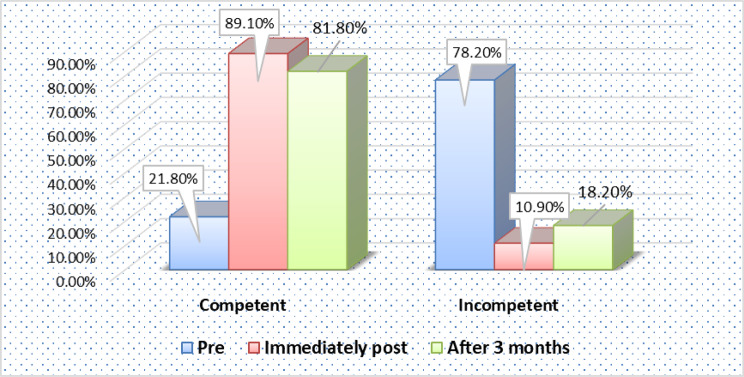
Parents’ total reported practice regarding care of their children after limb lengthening surgery pre, immediately post and after 3 months of nursing snippet-based intervention (*n* = 55)

Figure 1 portrays parents’ total reported practice regarding care of their children after limb lengthening surgery at pre, post and after 3 months of nursing snippet-based intervention implementation. It was found that, more than three quarters (78.2%) of the studied parent had incompetent reported practices pre- nursing snippet-based intervention. The majority of parents (89.1% and 81.8%) demonstrated competent reported practices at immediate post-test and at 3-month follow-up, respectively.

**Fig. 2:**
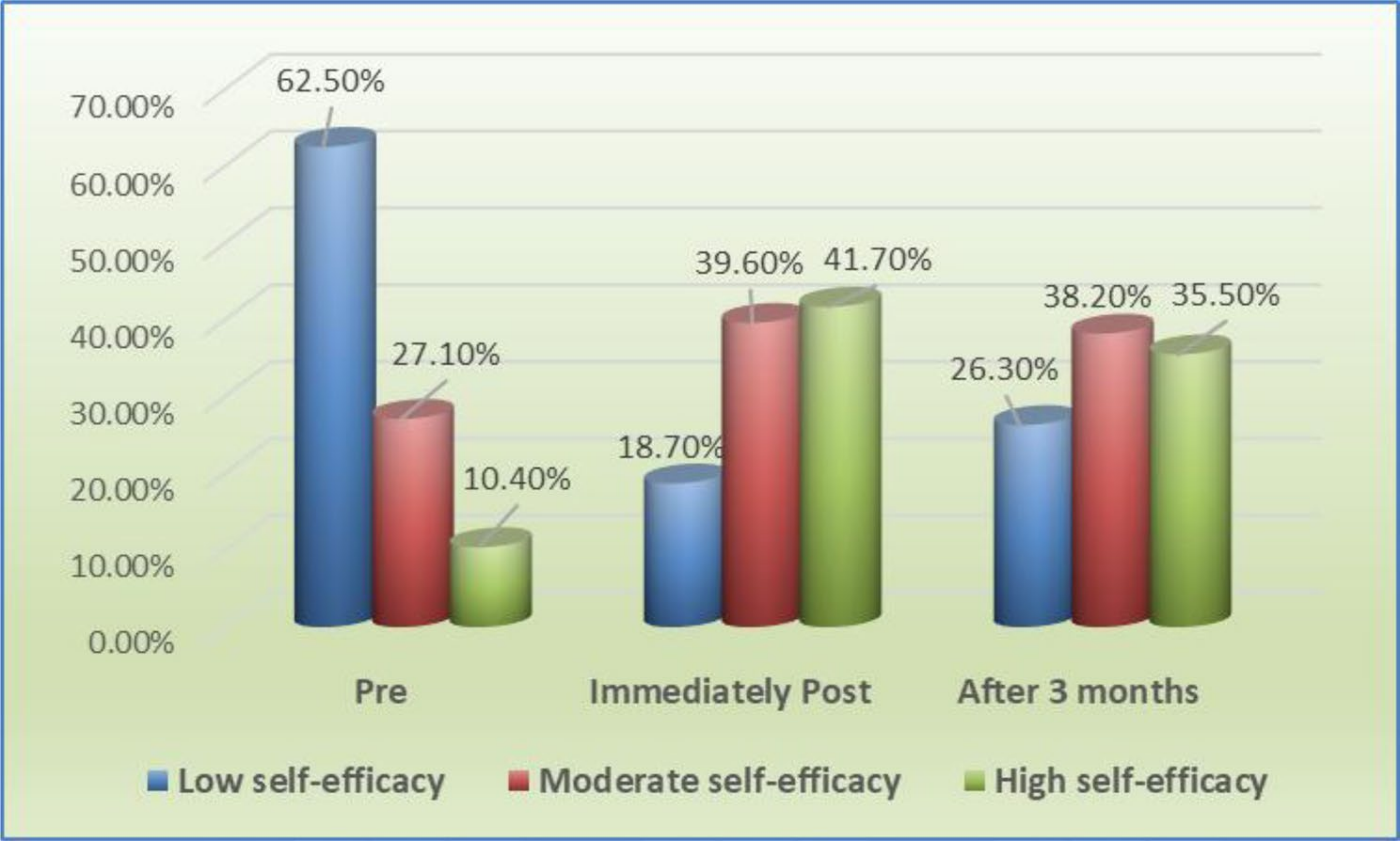
Parents’ total level of self-efficacy regarding care for their children after limb lengthening surgery pre, immediately post and after 3 months of nursing snippet-based intervention (*n* = 55)

Figure 2 shows Parents’ total level of self-efficacy regarding care for their children after limb lengthening surgery pre, immediately post and after 3 months of nursing snippet-based intervention. It was found that, less than two thirds (62.5%) of the studied parent had low self-efficacy pre- nursing snippet-based intervention. The proportion of parents with high self-efficacy was 41.7% at immediate post-test and 35.5% at 3-month follow-up.

Table 5 displays parents’ total burden levels at pre, immediately post and after 3 months of nursing snippet-based intervention; it was observed that (16.4% & 38.2%) had high and moderate burden level pre- nursing snippet-based intervention respectively. Low burden levels were reported among 40.0% of parents at immediate post-test and 36.4% at 3-month follow-up.

**Table 5:** Percentage distribution of parents’ total burden levels at pre, immediately post and after 3 months of nursing snippet-based intervention (*n* = 55)

Items	Pre a snippet-based teaching strategy implementation (n = 55)		Immediately post a snippet-based teaching strategy implementation (n = 55)		**After 3 months of** a **snippet-based teaching strategy implementation (n = 55)**		X2(1)	p-value	X2(2)	p-value
	No.	%	No.	%	No.	%				
**Parents’ total burden levels**							15.79	*p* < 0.001**	0.46	*p* > 0.927 ns
Little burden	7	12.7	22	40.0	20	36.4				
Mild burden	18	32.7	21	38.2	18	32.7				
Moderate burden	21	38.2	9	16.4	13	23.6				
High burden	9	16.4	3	5.5	4	7.3				

** Highly statistical significant difference at *p* value ≤ 0.001

^ns^ No statistical significant difference at *p* value *p* > 0.05

X2(1) Difference between pre-test and immediately post-test

X2(2) Difference between immediately post-test and follow-up test

Table 6 reveals correlation between total knowledge score, total reported practice score and total parents’ self-efficacy level of the studied parent pre, immediately post and after 3 months of nursing snippet-based intervention. A highly statistically significant positive correlation was observed between total knowledge scores, reported practice scores, and parents’ self-efficacy levels across pre-test, immediate post-test, and 3-month follow-up measurements.

**Table 6:** Correlation between total knowledge score, total reported practice score and total parents’ self-efficacy level of the studied parents pre, immediately post and after 3 months of nursing snippet-based intervention (*n* = 55)

Variables	Total knowledge		Total reported practices		Total parents’ self-efficacy level	
	Pre		Immediately post		After 3 months	
	r	p	r	p	r	p
**Total knowledge**	-	-	0.621	0.001**	0.538	0.001**
**Total reported practices**	0.452	0.001**	-	-	0.579	0.001**
**Total parents’ self-efficacy level**	0.385	0.005*	0.551	0.001**	-	-

**A highly statistical significant difference (*p* ≤ 0.001)

*A statistical significant difference (*p* ≤ 0.05)

r- Pearson Correlation Coefficient

## Discussion

The findings of the current study indicated that parents’ knowledge and reported practices showed higher post-test scores compared to pre-test measurements. Additionally, lower burden levels and higher self-efficacy scores were observed at post-intervention assessments. However, these results should be considered preliminary and non-causal due to the one-group pretest–posttest design and the lack of a control group. The possible common method bias resulting from single-session data collecting should also be taken into consideration when interpreting these results.

Due to their vulnerability, children and parents require close monitoring during the entire perioperative period. These findings highlight the importance of preoperative psychosocial assessment and continuous emotional support throughout healing and recovery. The best method for orthopedic surgeons to support children’s physical health and quality of life is to incorporate a parent’s self-efficacy oriented strategy into treatment plans [18].

Parents are important participants and practitioners in pediatric postoperative limb surgery [39]. Therefore, they should be able to provide extensive information to nurses on behalf of their children and provide timely feedback on the improvement of clinical interventions.

A nursing snippet-based intervention support parents were able to better understand caregiving processes without feeling overburdened by the information presented in brief, focused portions. This approach may be associated with reduced cognitive load and improved engagement, as information was presented in brief and focused segments which are frequently experienced by parents of children with complicated medical or surgical demands [40].

Related to complications that occurred as a result of limb lengthening surgery it was indicated that, two quarters had complications and less than half of them had joint stiffness. This result was agreed with [41] whose study entitled “calcaneal lengthening for correction of symptomatic flexible flat foot in children.” and revealed complications were reported in 17.5% on children. On the other hand, current study disagreed with [42] Whose study entitled “Results and complications of bilateral limb lengthening in achondroplasia: a retrospective analysis.” and demonstrated that a 4.7% increase in the risk of developing complications. Additionally, current study inconsistent with [43, 44]. Whose study entitled “Limb lengthening in achondroplasia” & “Bilateral double level tibial lengthening in dwarfism” and founded that, the complications were 33 out of 36 lengthened segments (92%), 28 out of 28 lengthened segments (100%), respectively. Also, in contrast, in the study by [45]. Whose study entitled “Femoral lengthening in achondroplasia: magnitude of lengthening in relation to patterns of callus, stiffness of adjacent joints and fracture.” all 20 patients reported temporary joint stiffness and fractures of bone regenerate occurred in 15% of cases. This discrepancy may be partially explained nursing snippet-based intervention, which placed a strong emphasis on early mobilization and preventive exercises.

As demonstrated, parents’ total knowledge regarding limb lengthening surgery pre, immediately post and after 3 months of nursing snippet-based intervention. It was found that, the majority of the studied parents had unsatisfactory knowledge pre- nursing snippet-based intervention. This result compatible with [46] whose study “Parental postoperative pain management perceptions, attitudes, and practices in pediatric limb fractures” and founded that, parents had insufficient knowledge about limb surgery, pain assessment tools, analgesics and causes of pain in children. This could be mainly attributed to negligence by health care professionals, unavailability of assessment tools, and inadequate limb surgery and pain related education.

The present study mentioned that, the majority of parents had competent total reported practice immediately post and after 3 months of nursing snippet-based intervention. This result competent with [47] which highlight the complex nature of the parental role of being a treatment supporter for a child with limb surgery, and observed that, the majority of parents performed a number of duties to support the treatment process. Unexpectedly from the parents’ point of view, they were given the role of an ‘extended arm’ of the healthcare team, in which they carried out direct and indirect treatment duties that were crucial to the treatment outcomes. From the researchers’ point of view, the snippet-teaching strategy provided parents with step-by-step, achievable learning units, enabling them to gain confidence in managing caregiving activities such as medication administration, wound care, and monitoring warning signs.

As regards parents’ total level of self-efficacy regarding care for their children after limb lengthening surgery, there were observed that, the majority of parents had high level of self- efficacy immediately post and after 3 months of nursing snippet-based intervention this result congruent with [18] whose study “Core psychosocial issues for children and adolescents in the context of limb lengthening and reconstruction surgery treatment” and showed, the majority of parents were weighted toward limb lengthening surgery and in the same time feel high self-efficacy after empowered with information about surgery. From researchers’ point of view, parents’ self-efficacy depends on understanding full picture about their children case.

According total parents’ burden levels, the current study demonstrated that, more than half of parents had moderate and high burden level pre- nursing snippet-based intervention implementation which congruent with [48] Whose study entitled “The factors associated with the caregiving burden among family caregivers of pediatric patients with fractures: A descriptive cross-sectional study” and founded that, most family caregivers were mothers comprising 56% of the total and 81.5% of caregivers had moderate or severe burden. From researchers’ point of view, this finding reflects that most participants were mothers, who are generally more emotionally responsive and more closely engaged in their children’s care. Also, increase parents’ burden linked to the lengthy course of limb lengthening therapy, which frequently entails numerous surgeries, multiple hospital stays, intense home care and ongoing condition monitoring. The simplicity, clarity, and accessibility of the nursing snippet-based intervention may be responsible for the decrease in parental burden seen in the current study.

Regarding correlation between parents’ total level of knowledge, total reported practices and their total self-efficacy level, there were highly statistical positive correlation between the studied parents’ total level of knowledge, total reported practices and their total self-efficacy level at pre, immediately post and after 3 months of a nursing snippet-based intervention. These results were consistent with [49]. Whose study entitled “Family caregiver strain and challenges when caring for orthopedic patients: a systematic review” and demonstrated, Orthopedic caregivers without enough information had less security in dealing with the children’ disease than those who received an appropriate education from health care team members: this is a finding that was consistent with the stress, self- efficacy and coping model of caregiving. This finding aligns with Bandura’s self-efficacy theory, which highlights how people’s perceived capacity to successfully carry out caregiving responsibilities can be diminished by a lack of knowledge and skills [23]. A slight decline in parents’ knowledge and practice was observed at three-month follow-up, which may indicate the need for ongoing reinforcement to maintain acquired competencies.

Regarding parents’ characteristics, the mean age of the studied parents was 35.94 ± 3.52 years. The majority were mothers, and more than two thirds resided in rural areas. In terms of educational level, more than two thirds had a university education. These findings are consistent with the study conducted by [47] titled “Swedish parents’ experiences of their role in treatment for children with congenital limb reduction deficiency: Decision-making and treatment support,” which reported a mean parental age of 40 years, that 70.5% of participants were mothers, and that 64.7% had university-level education. This particular demographic profile implies that, despite having the mental capacity to follow complicated instructions, these parents’ geographic location prevents them from receiving frequent in-person medical consultations. The snippet-based intervention served as a “digital lifeline” in this situation, connecting rural homes and metropolitan healthcare facilities. The solution reduced the need for difficult travel for minor clinical inquiries by empowering these educated but geographically remote parents to confidently undertake complex orthopedic treatment using bite-sized, easily available information via cellphones.

The predominance of mothers may be attributed to their primary caregiving role and responsibility in supporting their children’s health needs. Moreover, the combination of a high educational level and rural residence highlights a distinctive demographic profile that may influence access to continuous healthcare support and underscores the need for accessible digital health education strategies.

In addition, it was observed that all parents had not attended any previous training courses regarding limb lengthening surgery, indicating a critical gap in parental preparedness and emphasizing the urgent need for structured educational interventions. In this context, the nursing snippet-based microlearning approach may serve as a practical digital tool that bridges this gap by providing concise, accessible, and repeatable educational content, particularly for rural caregivers who may face challenges in repeated hospital visits. Accordingly, the intervention can be considered a mobile educational support strategy that associated with continuity of care beyond the hospital setting.

The current study showed that, mean age of children need limb lengthening surgery was 8.32 ± 2.53 year which inconsistent with the previous studies conducted by [50, 51], whose studies entitled “Quality of life evaluation following limb lengthening surgery in patients with achondroplasia” & “Comparison between upper and lower limb lengthening in patients with achondroplasia: A retrospective study.”, respectively and reported that, 12 children had received upper-and lower-limb lengthening; mean age at initial surgery 11.8 years with achondroplasia. The researchers reflect this as a result of differ sample size and setting of study.

## Conclusion of the study

The findings of the current study suggest that a nursing snippet-based intervention may be associated with improvements in parents’ knowledge, reported practices, and self-efficacy, as well as a potential reduction in perceived caregiving burden among parents of children undergoing limb lengthening surgery.

However, these results should be viewed cautiously and regarded as preliminary due to the lack of a control group and the methodological constraints of the study.

Although the findings suggest that nursing snippet-based microlearning might be a potential strategy, more research with more rigorous designs is required before conclusions about its efficacy or incorporation into standard pediatric orthopedic discharge education can be made.

### Recommendations

The current study recommends that future research further validate and scale up these findings through longitudinal studies and larger, more diverse samples across different settings. In addition, structured training programs for clinical staff should be developed to ensure fidelity and standardization in delivering the nursing snippet-based intervention. Furthermore, periodic booster sessions and continuous access to digital educational content are recommended to sustain long-term parental outcomes. Importantly, it is recommended that snippet-based education be integrated into routine discharge protocols for pediatric orthopedic surgeries, and that digital “snippet libraries” be established within hospital information systems as a sustainable educational resource rather than a one-time research-based intervention. Additionally, blended educational approaches, including printed illustrated materials and hospital-provided digital devices, may support equitable access for all caregivers.

### Limitations of the study

This study has several limitations. First, this study’s main limitation is the lack of a control group, which makes it difficult to conclusively link the intervention to the gains in parents’ knowledge and self-efficacy because other outside variables or time may have had an impact. Second, the findings’ generalizability may have been limited by the requirement that participants had a smartphone and be digitally literate, which may have excluded more vulnerable or lower-income parents who lack access to such technology (Selection Bias). Third, respondent weariness or common method bias may result from administering multiple questionnaires to the same participants, which could exaggerate the correlations between variables. Fourth, self-reporting was used to collect data on parents’ practices, which may include social desirability bias. Participants may indicate better practices than they actually engage in. Finally, the study may not be representative of all parents of children receiving limb lengthening surgery in various clinical or geographic situations because it was carried out in a single setting with a comparatively small purposive sample. Although the study identified significant associations between knowledge, practice, self-efficacy, and parental burden, other potentially influential variables such as clinical experience and personal motivation were not assessed and may have affected the results.

### The study’s consequences

Notwithstanding these limitations, the study offers initial proof of the viability and acceptability of microlearning (snippets) in pediatric orthopedic treatment, which will serve as a foundation for subsequent RCTs.

## Data Availability

The data and resources utilized in the current study cannot be made publicly available due to confidentiality considerations. On reasonable request, they can be obtained from the corresponding author.

## Abbreviations

CSES: Caregiver Self-Efficacy Scale
FGFR3: Fibroblast Growth Factor Receptor 3 gene
ZBI: Zarit Burden Interviewing
